# Biomarkers of Importance in Monitoring Heart Condition After Acute Myocardial Infarction

**DOI:** 10.3390/jcm14010129

**Published:** 2024-12-29

**Authors:** Aneta Aleksova, Alessandra Lucia Fluca, Antonio Paolo Beltrami, Elena Dozio, Gianfranco Sinagra, Maria Marketou, Milijana Janjusevic

**Affiliations:** 1 Cardiothoracovascular Department, Azienda Sanitaria Universitaria Giuliano Isontina, 34100 Trieste, Italy; alessandralucia.fluca@units.it (A.L.F.); gianfranco.sinagra@asugi.sanita.fvg.it (G.S.); mjanjusevic@units.it (M.J.); 2 Department of Medical Surgical and Health Sciences, University of Trieste, 34125 Trieste, Italy; 3 Dipartimento di Area Medica (DAME), Istituto di Patologia Clinica, University of Udine, 33100 Udine, Italy; antonio.beltrami@uniud.it; 4 Department of Biomedical Sciences for Health, University of Milan, 20122 Milan, Italy; elena.dozio@unimi.it; 5 Cardiology Department Crete, School of Medicine, Heraklion University General Hospital, University of Crete, 70013 Heraklion, Greece; maryemarke@yahoo.gr

**Keywords:** biomarkers, myocardial infarction, myocardial remodelling, heart failure, patient monitoring

## Abstract

Despite notable advancements in cardiovascular medicine, morbidity and mortality rates associated with myocardial infarction (MI) remain high. The unfavourable prognosis and absence of robust post-MI protocols necessitate further intervention. In this comprehensive review, we will focus on well-established and novel biomarkers that can provide insight into the processes that occur after an ischemic event. More precisely, during the follow-up, it is of particular importance to monitor biomarkers that indicate an increase in myocardial stretch and stress, damage and death of cardiomyocytes, remodelling of the extracellular matrix, oxidative stress, and inflammation. This enables the identification of abnormalities in a timely manner, as well as the capacity to respond promptly to any changes. Therefore, we would like to highlight the importance of well-known markers, such as natriuretic peptides, high-sensitivity troponins, soluble suppression of tumorigenicity 2, galactin-3, C-reactive protein, and interleukins in post-MI settings, as well as biomarkers such as adrenomedullin, growth differentiation factor-15, insulin-like growth factor binding protein 7, amyloid beta, vitamin D, trimethylamine N-oxide, and advanced glycation end-products that recently emerged in the cardiovascular filed. The implementation of novel post-MI protocols, which encompass the monitoring of the aforementioned biomarkers deemed pertinent, in conjunction with adherence to established cardiac rehabilitation programmes, along with the already well-established therapeutic strategies and control of cardiovascular risk factors, has the potential to markedly enhance patient outcomes and reduce the elevated level of morbidity and mortality.

## 1. Introduction

Acute myocardial infarction (MI) remains a significant global health burden, despite medical advances in terms of improved reperfusion techniques and appropriate medical therapy that have greatly improved patient survival rates. Indeed, the incidence of the disease and the risk of morbidity and mortality remain persistent [1]. More precisely, patients who have experienced an acute MI are at high risk of developing further complications, including recurrent MI, arrhythmias, and cardiac remodelling that may lead to a general decline in cardiac function and may ultimately result in heart failure (HF) and death [2].

The current standard post-MI protocol includes regular follow-ups, therapeutic interventions, and patient counselling on lifestyle changes that can decrease the risk of future cardiac events. Unfortunately, in practice, it has been demonstrated that patients are often less adherent to these recommendations, which may ultimately limit their effectiveness [3]. It is therefore of the utmost importance to develop innovative protocols that would encourage not only patients, but also healthcare professionals, to provide additional counselling on risk factor mitigation, improve patient awareness of their condition and potential outcomes, provide psychosocial support, and explain cardiac rehabilitation programmes in detail. Indeed, cardiac rehabilitation represents a promising approach that has been demonstrated to reduce cardiac remodelling, the incidence of recurrent events, and the development of HF [4]. It is unfortunate that cardiac rehabilitation is underutilised due to a combination of factors, including a lack of awareness among patients of the existence of such programmes and their benefits, as well as a shortage of knowledge, expertise, and interest among healthcare professionals themselves coupled with limited resources within hospitals [5].

Finally, multiple studies have demonstrated that a vast number of biomarkers may facilitate the diagnosis and close monitoring of patients who have survived a MI, thereby offering insights into the underlying processes that occur following an ischemic event [6]. According to the Food and Drug Administration and the National Institutes of Health (FDA-NIH) Biomarker Working Group, a biomarker is “a defined characteristic that is measured as an indicator of normal biological processes, pathogenic processes, or responses to an exposure or intervention” [7]. A general interest in identifying novel biomarkers to better diagnose diseases, prognostically stratify patient outcomes, and follow the evolution of pathologies is relevant. This concept is supported by important investments that have been made in the field of biomarker discovery over the last three decades. However, despite this, only a few biomarkers have proven to be clinically useful and are currently being employed [8]. Several possible explanations for this discrepancy have been identified, but one of the most important is that a biomarker should be useful in addressing carefully defined, clinically relevant questions, adding more information than what is already available [8,9]. A second explanation concerns the study design for biomarker identification, which usually employs a “triangular” strategy, with a discovery phase conducted on a few patients, followed by verification and validation phases conducted on thousands of patients. Most of the studies that are being conducted reach only the discovery phase and do not progress to validation, thus reducing the clinical impact of the research [10]. The use of high-throughput omics techniques coupled with artificial intelligence approaches holds the promise of improving the identification of new biomarkers in the future by means of “rectangular strategies” [10,11].

With regard to patients affected by cardiovascular disease (CVD), the two main clinical questions that have been addressed in this review are related to the diagnosis of acute MI and HF. In the latter case, the most frequent application is to rule out the diagnosis of HF in clinical scenarios where it is in the differential (e.g., patients with dyspnoea). Following an insult such as MI or hemodynamic changes, the presence of inflammation and/or altered neurohormonal activation, cardiac muscle undergoes a series of structural and functional processes collectively referred to as cardiac remodelling [12]. Consistently, HF biomarkers have been grouped based on the main pathophysiological alterations occurring in this syndrome, such as myocardial stretch and stress, cardiomyocyte damage and death, remodelling of the extracellular matrix, oxidative stress, and inflammation (Table 1) [9]. Among all available biomarkers, those that are most commonly employed in clinical practice for the monitoring of cardiac remodelling are natriuretic peptides, high-sensitivity troponins, soluble suppression of tumorigenicity 2 (sST2), and galactin-3 (Gal-3) [12,13].

Therefore, in this comprehensive review, we will discuss well-established old and new molecules that can enhance patient diagnosis and monitoring after an MI, provide more detailed insights into processes such as left ventricular dysfunction and adverse remodelling [14], and the impact of minor cardiac alterations that could result in significant complications over time, such as HF and death.

**Table 1:** Summary of biomarkers divided according to pathophysiological alterations in heart failure.

Pathophysiological Alterations	Biomarkers	References
Myocardial stretch/stress	BNP and NT-proBNP	[13]
	sST2	[13,15]
	Adrenomedullin	[16,17]
	GDF-15	[18,19]
	IGFBP7	[20,21,22]
Cardiomyocyte death	Troponins	[23]
Remodelling and fibrosis	Metalloproteases and inhibitors of metalloproteases	[9]
	Pro-collagen I C telopeptide:MMP1	[24]
	Gal-3	[13,15]
Inflammation	C-reactive protein	[25]

BNP, B-type natriuretic peptide; NT-proBNP, N-terminal pro-B-type natriuretic peptide; Gal-3, galactin-3; GDF-15, growth differentiation factor-15; IGFBP7, insulin-like growth factor binding protein 7; MMP1, metalloprotease 1; sST2, soluble suppression of tumorigenicity 2.

## 2. Biomarkers

### 2.1. Biomarkers of Myocardial Stretch/Stress

Natriuretic peptides are powerful biomarkers recommended by the latest European and American HF guidelines, with well-defined cut-off values for the assessment of acute and chronic HF, including brain or B-type natriuretic peptide (BNP) and its inactive form N-terminal pro-B-type natriuretic peptide [13]. BNP is a hormone produced by myocytes in response to stretch, which may be caused by volume or pressure [13], and elevated values of this marker post-MI are associated with adverse remodelling, including left ventricular dilation, hypertrophy, and decreased systolic function. Furthermore, elevated BNP levels post-MI or in chronic HF patients are associated with a higher risk of adverse outcomes. Consequently, by monitoring BNP, healthcare professionals are able to identify early indications of HF, better stratify the risk, and personalise treatments to enhance outcomes in patients after an MI [12,13]. Here, it is important to mention that the administration of an angiotensin receptor–neprilysin inhibitor (ARNI), such as sacubitril/valsartan, is recommended in patients at risk of adverse cardiac remodelling and HF post-MI [26], and in cases of already established HF with reduced ejection fraction as a result of MI [27]. The PARADIGM-HF Trial reported a significant increase in BNP levels shortly following sacubitril/valsartan administration [28]. Indeed, the inhibition of neprilysin, which is responsible for the degradation of BNP, results in an increase in this biomarker [27]. Therefore, this has raised questions about the effectiveness of BNP in reflecting the risk/state of HF. However, the authors of the PARADIGM-HF Trial indicate that both BNP and NT-proBNP remain valuable in monitoring risk in patients with HF, even though they are treated with sacubitril/valsartan [28].

sST2 is a decoy receptor of IL-33 that is released in conditions of myocardial and vascular strain. IL-33 is a biomarker with important cardioprotective features, including the prevention of cardiac fibrosis and hypertrophy. It has been demonstrated that, following the binding with sST2, the aforementioned processes are inhibited [13]. Consequently, numerous studies noted elevated levels of circulating sST2 in patients after MI, chronic HF, and/or with hypertension. Furthermore, sST2 has been observed to have a strong ability to indicate the early stages of cardiac remodelling and hypertrophy. Therefore, serial measurement of this biomarker during follow-up after an MI event can provide significant input in terms of patient outcome [15].

A further biomarker related to cardiac volume overload is the hormone adrenomedullin (ADM), whose physiological function is to maintain endothelial barrier function and to inhibit the renin–angiotensin–aldosterone system [16]. ADM is secreted as a pre-pro-hormone. Following the removal of the signal peptide by a peptidase, the pro-hormone is further processed by a convertase to generate four peptides: proadrenomedullin NH2-terminal (PAMP), midregional proadrenomedullin (MR-proADM), C-terminally glycine-extended adrenomedullin (Adrenomedullin-Gly), and C-terminal proadrenomedullin (CT-proADM), also known as adrenotensin [17]. Adrenomedullin-Gly is inactive and is converted into the active form following C-terminal amydation (bio-ADM) [17]. Given the short half-life of ADM and the presence of a binding protein, the MR-proADM has been analysed for diagnostic purposes as a proxy for bio-ADM [29], with few exceptions [17]. ADM is elevated in patients with sepsis and acute HF. Several studies claim that MR-proADM is an independent prognostic biomarker of cardiac dysfunction, associated with hemodynamic impairment and tissue congestion, that is able to predict the mortality risk of patients with HF, even more accurately than natriuretic peptides. However, other studies have not corroborated these findings [30]. Ongoing studies are also verifying the therapeutic potential of ADM, given its positive inotropic effect [30]. Ongoing studies are also verifying the therapeutic potential of ADM, given its positive inotropic effect [30].

Growth differentiation factor-15 (GDF-15) is a member of the transforming growth factor-β superfamily that is expressed at very low levels in the tissues of healthy individuals (with the exception of the placenta). Since its gene promoter contains two p53 binding sites, it is highly induced in response to hypoxia, inflammation, oxidative stress, and DNA damage [18,19]. GDF15 is produced by adipose tissue, atherosclerotic plaques, the myocardium and peripheral tissue. In spite of this lack of specificity, GDF15 is emerging as a very interesting biomarker for cardiovascular risk in community-dwelling individuals, whereas low GDF15 levels are associated with longevity [18]. In individuals with coronary artery disease and HF, GDF15 seems to be more associated with mortality and disease development than classic risk predictors [18].

Another marker related to cell stress is insulin-like growth factor binding protein 7 (IGFBP7). This factor has been identified as one of the core components of the secretome of cells undergoing cellular senescence (i.e., a complex response to a variety of stressors that determines the permanent withdrawal of the cell from the cell cycle and promotes profound changes in gene expression, with upregulation of genes coding for secreted molecules). IGFBP7 seems to play a pathophysiological role in HF, where the accumulation of senescent cells is well known to occur. Indeed, IGFBP7 deficiency attenuates inflammation, cardiac fibrosis, and cell senescence in the hearts of mice exposed to transverse aortic constriction [31]. From a clinical standpoint, the circulating levels of IGFBP7 are increased in patients suffering from HF, are associated with diastolic function in HF patients with either normal or reduced ejection fraction [20,21,22], and predict risk for hospitalisation or death and renal outcomes [32].

### 2.2. Cardiomyocyte Death Biomarkers

Troponins are structural components of striated muscles that regulate contraction at the sarcomeric level. Specifically, in sarcomeres, troponin is a complex formed by the three subunits (T, C, and I), that undergoes a conformational change in response to an increase in intracellular calcium and regulates the interaction between actin and myosin, thus coupling excitation with contraction. Upon cardiomyocyte death, troponins are released from cardiomyocytes to the extracellular fluid and the blood. Cardiac-specific troponin isoforms have been used to identify cardiomyocyte necrosis since the 1980s; however, technical advancements in the analytical instruments have produced assays that have three specific characteristics: they can dose cardiac troponins (both T and I) in the ng/L range, they can detect troponin levels above the limit of detection in at least 50% of healthy individuals, and they have a total imprecision at the 99th percentile of the reference interval <10% [23]. The introduction of high-sensitivity troponin (hsTn) assays has been a paradigm shift, and has increased the identification of patients with ongoing chronic myocardial injury in patients without acute coronary syndromes [33]. This finding is indicative of ongoing cardiomyocyte loss, predicts adverse remodelling, and enables precise quantification of the severity of the patient’s condition [12]. Indeed, elevation of hsTn above the 99th percentile is associated with increased mortality [33].

### 2.3. Biomarkers of Extracellular Matrix Remodelling and Fibrosis

The process of extracellular matrix remodelling is the result of a balanced action between matrix metalloproteases (MMPs) and tissue inhibitors of metalloproteases (TIMPs). Fibrosis occurs when the action of these enzymes is altered or the extracellular matrix is chemically modified by non-enzymatic glycation (see discussion below) or excessively crosslinked [34,35]. From a clinical point of view, MMP2, MMP9, and TIMP-1 levels have been associated with mortality in patients affected by HF [9]. Furthermore, biomarkers of collagen turnover, such as the C-terminal telopeptide (CTX), a fragment of Type I collagen that is released into the blood following its degradation, have been associated with cardiovascular deaths/HF in patients suffering from NSTEMI, independently from cardiac troponin, NT-proBNP, and C-reactive protein levels [36]. In this regard, a very interesting biomarker is the ratio between pro-collagen I C telopeptide, which may be viewed as an indicator of collagen cross-linking and resistance to proteolytic degradation. Clinically, patients with a high CITP:MMP1 ratio (i.e., less collagen cross-linking) are more sensitive to the anti-fibrotic effects of aldosterone receptor blockade [24].

Another marker associated with cardiac fibrosis is Gal-3. Although Gal-3 is not an organ-specific marker, it has been observed that elevated levels of Gal-3 are associated with increased inflammation, atherosclerosis, hypertrophy, and fibrosis. Furthermore, as with sST2, serial measurements of Gal-3 can assist in the identification of early stages of cardiac remodelling. Additionally, this biomarker has been demonstrated to possess significant prognostic value with regard to mortality risk in patients with cardiac diseases [13,15].

### 2.4. Biomarkers of Inflammation

As anticipated, inflammation plays a very relevant pathophysiological role. C-reactive protein (CRP), a member of the pentraxin family of innate immunity proteins, is secreted both by the liver in response to Interleukin (IL)-6 and by smooth muscle cells, especially in atherosclerotic vessels [37]. On top of being a biomarker for inflammation, CRP plays a role in atherosclerosis development [38]. Owing to the development of high-sensitivity tests, it has been possible to identify that, even when low levels of this protein are quantified, these are endowed with prognostic significance [39]. Indeed, CRP levels < 1, 1–3, and >3 mg/L identify patients at low, intermediate, and high risk for CVD [25]. Moreover, in patients affected by chronic HF, CRP levels assessed by high-sensitivity assays are associated with all-cause and cardiovascular mortality [40]. Furthermore, the persistence of the inflammatory response, assessed as the area under the curve of repeated CRP assessments is associated with mortality in the 3 years following discharge of patients hospitalised for acute HF [41]. CRP has been considered to be a surrogate for the activity of IL-1, an interleukin whose pathophysiological role in HF is becoming more and more apparent. Indeed, its blockade via Anakinra reduces HF-related mortality [41]. Although IL-1 plasma levels are associated with the outcome of patients affected by HF [42], cytokine concentrations are very low (in the pM range) and these analytes are very temperature-sensitive [43,44]. For this reason, alternative analytes (e.g., CRP, IL-6, soluble urokinase plasminogen receptors) have been tested for their ability to better assess the inflammatory burden in patients affected by HF [45,46].

### 2.5. Emerging Biomarkers in the Cardiovascular Field

In addition to these well-established biomarkers, it is worth mentioning here some of the less well-known ones that are emerging in this clinical setting. As anticipated, the number of candidate biomarkers, associated with different pathogenetic processes of heart disease that have the potential to assist in patient monitoring is considerable, largely owing to the use of high-throughput omics platforms. Given the vast number of candidates that could be relevant for patient monitoring, it is not feasible to include them all in this review; however, we are going to mention some of them that we have deemed worthy of further consideration.

One of the biomarkers that is undoubtedly gaining attention in the cardiovascular field is amyloid beta 1-40 (Aβ1-40), due to its pro-inflammatory and pro-atherosclerotic role, and its association with poor outcomes in patients with CVD [47,48]. For a long time, Aβ peptides have been intensely investigated for their pathogenetic role in diseases of the brain and associated vasculature. Subsequently, it was observed that Aβ-40 is localised in the heart and in the vascular system, particularly in the wall of the carotid artery, the aorta, and the coronary arteries; that a considerable amount of Aβ1-40 is produced by platelets within plaques; and that there is a high association between Aβ1-40 levels and MACE [47,48,49]. Therefore, this molecule could be of significant utility for patient follow-up. More precisely, Aβ1-40 is involved in plaque formation and destabilisation, and it acts as an inflammatory stimulant, activating monocytes and resulting in a significant increase in tumour necrosis factor α (TNF-α) and MMP-9 production [48,49]. Furthermore, elevated Aβ1-40 levels have been associated with arterial stiffness and are also implicated in the aetiology of atrial fibrillation [49].

Therefore, the administration of targeted medications to reduce Aβ1-40 levels in patients with acute MI could be a promising approach to improving patient prognosis. It has been demonstrated that cholesterol-lowering drugs reduce the production of Aβ1-40 by influencing the activities of amyloid precursor protein-secretase [50]. Furthermore, certain cardiovascular drugs with antihypertensive properties, such as β-blockers or calcium channel receptor antagonists, are effective in reducing the concentration of Aβ1-40 [51]. In this context, it is important to note that certain drugs that are in use to treat patients with CVD may exhibit beneficial effects targeting specific pathways, while on the other hand, they may also have adverse effects on other aspects of health. For instance, angiotensin-converting enzyme inhibitors are commonly used because of their beneficial effects on the clinical outcomes of patients with CVD. However, these inhibitors also appear to promote Aβ1-40 production [51]. In addition, the chronic use of sacubitril/valsartan results in the accumulation of Aβ1-40 owing to its impaired clearance [52]. Lastly, numerous studies have demonstrated that physical activity has a positive effect on cognitive improvement in patients with Alzheimer’s disease, reducing Aβ levels [53,54]. In particular, regular physical activity reduces oxidative stress, neuronal cell death, and the production of pro-inflammatory markers such as TNF-α and IL-1α, thereby decreasing Aβ accumulation. Furthermore, physical activity has been shown to increase endothelial nitric oxide synthase (eNOS) activity in the brain vasculature [53,54], a molecule with multiple roles in maintaining cardiovascular health [55]. However, the impact of physical activity on Aβ molecules outside Alzheimer’s disease remains limited, and it could be only assumed that exercise might have a similar effect on Aβ production and accumulation outside the brain system.

Another biomarker recognised to be extremely important in the cardiovascular field is vitamin D [56]. Vitamin D has emerged as a cardioprotective and prognostic biomarker due to its multiple roles in processes such as the regulation of inflammation, oxidative stress, mitochondrial respiratory function, and calcium and glucose homeostasis [57,58,59]. Vitamin D promotes an anti-inflammatory state by reducing the production of pro-inflammatory cytokines including TNF-α, IL-1β, and IL-6 through the inhibition of nuclear factor kappa light chain enhancer of activated B cells (NF-κB) activity [57]. Therefore, as a consequence of hypovitaminosis D, the increase in TNF-α transcription leads to a reduction in the nitric oxide (NO) concentration and oxidative stress, resulting in inflammation and endothelial dysfunction, which could lead to the development of atherosclerosis. Vitamin D is also involved in the up-regulation of numerous antioxidants besides anti-inflammatory cytokines, maintenance of mitochondrial function, and regulation of reactive oxygen species (ROS) levels. If the concentration of this vitamin is not optimal, vitamin D cannot alleviate oxidative stress, leading to increased intracellular oxidative damage, aberrant cell proliferation, and elevated levels of apoptosis [58]. In addition, vitamin D is involved in the regulation of the renin–angiotensin–aldosterone system (RAAS) by suppressing renin and angiotensinogen genes through various mechanisms, thereby regulating blood pressure [59].

Finally, several studies have examined the relationship between hypovitaminosis D and cardiac remodelling in patients with MI. The proposed scenario involves multiple pathways, including the loss of vitamin D regulation of inflammation, increased production of MMP-9, and elevated RAAS activity, which lead to cardiac hypertrophy and fibrosis through distinct, but highly intertwined, mechanisms [59]. Therefore, the optimisation of vitamin D levels through a diet including food fortified with this hormone, sun exposure, and supplementation should be considered as a preventive measure, especially in the post-MI scenario [14]. However, caution is needed in view of the fact that optimal levels of vitamin D are beneficial, but excessive levels of this soluble hormone are toxic and associated with adverse outcomes [60,61]. Furthermore, despite the proven association between hypervitaminosis D and MACE, there is a great deal of inconsistency regarding vitamin D supplementation in clinical trials, suggesting that further interventional studies are needed [59,62].

Following the previous points regarding diet, it might be helpful to mention here a biomarker that is largely influenced by diet and is highly associated with adverse outcomes in patients with MI [63]. Trimethylamine N-Oxide (TMAO) is a microbiota-dependent metabolite that has recently been shown to play a role in the development and progression of atherosclerosis [63]. The consumption of foodstuffs such as dairy products, red meat, egg yolk, and fish rich in choline, L-carnitine, and phosphatidylcholine has been shown to increase the risk of elevated levels of this metabolite. Fish have historically been regarded as beneficial for cardiovascular health. However, recent findings have indicated that an abundance of substrate that ultimately increases the TMAO concentration may have a contrary effect [63]. Further studies are required to elucidate the conflicting data. Nevertheless, it is important to note that elevated levels of TMAO are associated with the development of inflammation and oxidative stress in the body, which in turn can lead to the advancement and worsening of atherosclerosis and the occurrence of adverse cardiac ischemic events during follow-up in the setting of a previous MI [63]. In our recent study, we noted for the first time that the in-hospital fluctuation of TMAO after an MI event has a significant impact on patient clinical outcomes. More precisely, we conducted a continuous hazard ratio analysis to determine a cut-off value of 3.45 mM for TMAO. Our findings indicated that TMAO values higher than the cut-off were linearly associated with an increased risk of adverse outcomes during follow-up. Furthermore, we observed that patients with MI and persistently elevated TMAO levels or those with low TMAO levels at the time of their admission to the hospital, but whose levels subsequently increased beyond the median level of 3.45 mM during their hospitalisation, exhibited a two-fold increased risk of adverse outcome compared with patients with low or decreasing TMAO levels [63].

It is also worth mentioning the biomarkers fibroblast growth factor (FGF)-23 and its co-receptor klotho [64]. FGF-23 and vitamin D are pivotal regulators in mineral metabolism, particularly in the context of phosphate and calcium homeostasis [65]. The relationship between these two factors is intricate and involves a feedback loop. FGF-23 exerts a negative regulatory effect on vitamin D activation, while vitamin D stimulates FGF-23 expression [65]. Elevated FGF-23 levels have been associated with hypertension, hypertrophy, and HF, and several studies have noted significant prognostic potential regarding mortality risk during long-term follow-up. Klotho, on the other hand, exerts cardioprotective effects, including the regulation of endothelial function by regulating NO activity and the suppression of oxidative stress and inflammation. Therefore, it is not surprising that reduced klotho levels are associated with an inflammatory state and atherosclerosis [64,66]. Furthermore, klotho deficiency indicates mortality risk in patients and it has been argued that novel therapeutic approaches should concentrate on activating klotho activity [66]. Interestingly, a recent study demonstrated that patients with MI who underwent the cardiac rehabilitation program had a higher increase in klotho levels compared with patients who did not adhere to such a program, indicating the beneficial effects of exercise [64], providing further evidence at the molecular level that supports the exercise component of cardiac rehabilitation.

**Figure 1:**
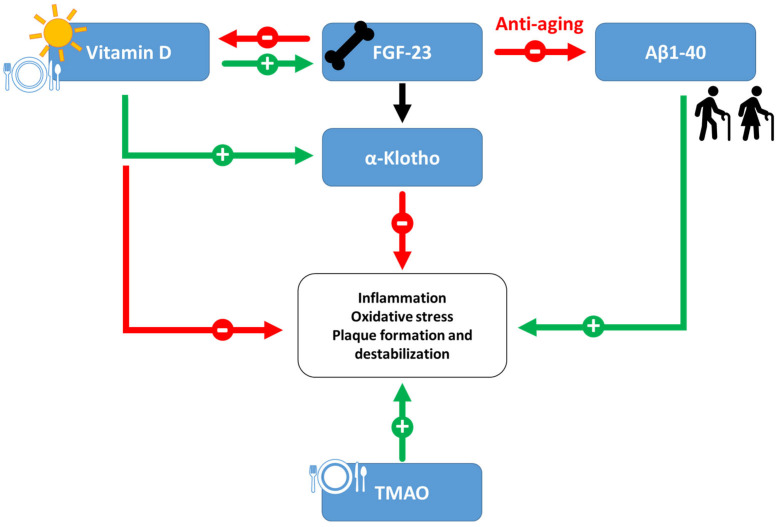
Overview of emerging cardiovascular biomarkers. Vitamin D deficiency, Klotho downregulation, heightened FGF-23 levels, beta-amyloid, and TMAO accumulation lead to elevated levels of inflammation, oxidative stress, plaque formation, and destabilisation.

Figure 1 provides a summary of the role of the emerging biomarkers discussed thus far, illustrating their key mechanisms and interactions in the context of cardiovascular pathophysiology.

**Figure 2:**
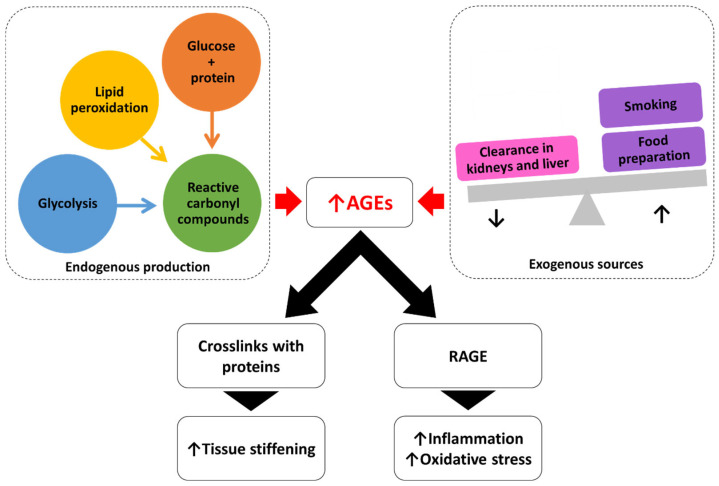
Endogenous production and exogenous sources of AGEs. On the one hand, the accumulation of AGEs leads to crosslinks with proteins contributing to tissue stiffening. On the other hand, AGEs enhance inflammation and oxidative stress after binding to RAGE. AGEs, advanced glycation end products; RAGE, receptors for advanced glycation end products.

In the context of novel biomarkers, it is worth mentioning here the advanced glycation end products (AGEs), heterogeneous compounds that result from the reactions of glycation, carbonylation, and oxidation of numerous substrates. The most commonly known AGEs are produced by the Maillard reaction between sugars, typically glucose, and proteins, which generates a Schiff base following the formation of a more stable compound consisting of Amadori products that are oxidised to form the final AGEs. Glycated haemoglobin is a common example of this type of reaction [67]. Alternatively, lipid peroxidation through interactions with ROS is similarly implicated in the formation of AGEs [67]. Finally, the glycolysis pathway is responsible for the production of carbonyl compounds that interact with proteins, resulting in the formation of AGEs [67]. In addition to the endogenous production of AGEs, certain habits can enhance the production of such compounds. Smoking is a well-documented source of AGEs, as is the consumption of food products prepared at high temperatures, and AGEs accumulate due to impaired clearance in the kidneys and liver [68]. The detrimental effects of AGEs are mediated by two distinct mechanisms. On the one hand, AGEs cause the formation of crosslinks with proteins such as elastin and collagen, which results in the stiffening of heart and vascular tissues [67]. Therefore, AGEs lead to diastolic and endothelial dysfunction [67]. Moreover, AGEs can bind to receptors for advanced glycation end-products (RAGEs), a class of membrane receptors belonging to the immunoglobulin family, triggering the activation of secondary messengers, including NF-κB and phosphoinositide 3-kinases (PI3Ks), which are mediators of oxidative stress and pro-inflammatory status [68]. Several seminal studies have highlighted the potential concerns regarding the role of RAGE in the cardiovascular field. These studies have indicated that these receptors may trigger pro-atherosclerotic and pro-inflammatory pathways [69] (Figure 2).

From a clinical perspective, these alterations are evaluated using the coronary artery calcium score, increase in tunica intima stiffness, and 18F-fluorodeoxyglucose-positron emission tomography [67]. It is noteworthy that numerous ligands, in addition to AGEs, can activate RAGE, including High-Mobility Group Box 1 (HMGB1), Aβ peptides, and S100/calgranulin [68]. In addition to the transmembrane form of RAGE, a soluble form of RAGE (sRAGE) has been identified in plasma, cerebrospinal fluid, and synovial fluid [69]. Two alternative pathways are involved in the production of sRAGE, namely the proteolytic cleavage of transmembrane RAGE and the alternative splicing that originates from a protein lacking the transmembrane and intracellular domains [69]. In particular, sRAGE acts as a decoy receptor that modulates the inflammatory response. The levels of sRAGE have been evaluated in patients with ACS, with contrasting results observed when compared with healthy controls as for the association of sRAGE with the outcome [70]. Finally, sRAGE and AGEs levels were evaluated in patients with acute MI before and after a three- to four-week cardiac rehabilitation programme [71]. Interestingly, lifestyle changes have been demonstrated to be an effective strategy to increase sRAGE levels and decrease AGEs concentrations, thus opening an interesting scenario for further investigation into the advantages of cardiac rehabilitation.

## 3. Current Approach in Biomarker Use in Patients with MI and Post-MI

In the acute phase of MI, the primary objective in clinical practice is to assess the extent of myocardial injury and the immediate damage to the heart muscle. HsTn is the gold-standard biomarker in this context because its levels rise rapidly after the onset of MI and can provide immediate confirmation of ongoing myocardial injury [2,72]. However, although BNP or NT-proBNP are crucial for assessing HF [13], they can also be employed in the context of MI due to their ability to indicate acute cardiac stress, particularly in patients with complications such as HF [2]. Similarly, sST2 can be utilised as a diagnostic tool due to its ability to reflect myocardial stress and injury [15]. Of greater significance, however, is its capacity to identify patients at risk of developing complications such as HF [13]. It is worth mentioning here that more studies are needed to establish the cut-off value of sST2 in acute MI settings.

In the early post-MI period, the focus shifts to monitoring the recovery process, assessing cardiac remodelling and the risk of complications such as arrhythmias, recurrent cardiac events, and the development of HF. Markers of interest in current clinical practice are still BNP or NT-proBNP for assessing the risk of HF, as well as markers in lipid profile and CRP [73]. Since the remodelling process following MI is driven by inflammation, monitoring CRP and interleukins (interleukins 1 and 6) could be informative in guiding treatment in the context of residual risk [2]. However, the current European guidelines lack precise indications regarding the integration of inflammation biomarkers into the clinical decision-making process. This is due to the necessity for further investigating novel biomarkers for the long-term management of patients who have experienced an MI [2]. In this context, GDF-15 has also been shown to contribute to inflammation, which is associated with poorer outcomes [18]. However, it is important to highlight that GDF-15 clinical practice is not currently recommended by major guidelines, as its role remains primarily investigational [74]. A comparable conclusion can be drawn with regard to Gal-3. Although this marker is of great importance and provides valuable insight into cardiac remodelling, worsening cardiac function, and mortality, its role remains primarily of an investigational nature and is not included in the official guidance for post-MI settings [13]. It is, however, included in HF settings [13].

Other biomarkers that have been shown to be highly informative in the post-MI setting and that have been discussed in detail in the previous part of this review, such as vitamin D and FGF-23, α-Klotho, TMAO, Aβ1-40, AGEs, adrenomedullin, and IGFBP7, remain in the research field. The informative value provided by these markers could be assessed by practitioners, although the currently available data are insufficient to establish a definitive protocol for the use of these biomarkers in the management of patients with acute and post-MI. Further research is required to establish suitable cut-offs for these biomarkers.

In conclusion, with regard to the acute MI and post-MI protocols, it can be stated that, at the present time, only troponins, BNP, and NT-proBNP can be employed in clinical practice due to the established cut-offs, as well as optimal vitamin D values (30–60 ng/mL) [75]. Optimal vitamin D levels are recommended as a preventive strategy, rather than the therapeutic solution for new ischemic events and mortality. With regard to other biomarkers, although they show considerable promise in the diagnosis and monitoring of MI and in guiding treatment decisions and improving patient outcomes by identifying those at higher risk of complications and poor recovery, further studies are required.

## 4. Conclusions

Despite medical advances, patients who survive acute MI remain at high risk of morbidity and mortality. Consequently, there is a pressing need for the development of improved post-MI protocols that could enhance patient outcomes and facilitate patient follow-up. A number of biomarkers can be employed to fulfil this function, offering insight into processes such as left ventricular dysfunction and adverse remodelling. In particular, biomarkers that indicate increased myocardial stretch and stress, cardiomyocyte damage and death, extracellular matrix remodelling, oxidative stress, and inflammation should be monitored on a regular basis and their values analysed during patient follow-up. This allows for the identification of abnormalities in a timely manner, as well as the ability to react promptly to any changes. Finally, the implementation of comprehensive monitoring of the biomarkers of interest in conjunction with cardiac rehabilitation protocols, coupled with the already well-established therapeutic strategies and control of cardiovascular risk factors, may significantly reduce the high levels of morbidity and mortality currently observed among patients who have survived MI.
